# Efficient Resources Provisioning Based on Load Forecasting in Cloud

**DOI:** 10.1155/2014/321231

**Published:** 2014-02-20

**Authors:** Rongdong Hu, Jingfei Jiang, Guangming Liu, Lixin Wang

**Affiliations:** ^1^School of Computer, National University of Defense Technology, Changsha 410073, China; ^2^National Supercomputer Center, Tianjin 300457, China

## Abstract

Cloud providers should ensure QoS while maximizing resources utilization. One optimal strategy is to timely allocate resources in a fine-grained mode according to application's actual resources demand. The necessary precondition of this strategy is obtaining future load information in advance. We propose a multi-step-ahead load forecasting method, KSwSVR, based on statistical learning theory which is suitable for the complex and dynamic characteristics of the cloud computing environment. It integrates an improved support vector regression algorithm and Kalman smoother. Public trace data taken from multitypes of resources were used to verify its prediction accuracy, stability, and adaptability, comparing with AR, BPNN, and standard SVR. Subsequently, based on the predicted results, a simple and efficient strategy is proposed for resource provisioning. CPU allocation experiment indicated it can effectively reduce resources consumption while meeting service level agreements requirements.

## 1. Introduction

Cloud computing offers near-infinite amount of resources capacity (e.g., CPU, memory, Network *I/O*, disk, etc.) at a competitive rate and allows customers to obtain resources on-demand with pay-as-you-go pricing model. Instead of incurring high upfront costs in purchasing Information Technology (IT) infrastructure and dealing with the maintenance and upgrades of both software and hardware, organizations can outsource their computational needs to the cloud. The proliferation of cloud computing has resulted in the establishment of large-scale data centers containing thousands of computing nodes and consuming enormous amounts of electrical energy.

According to previous studies in the past decade, the reason for this extremely high energy consumption is not just the quantity of computing resources and the power inefficiency of hardware but rather the inefficient usage of these resources. The data collected from more than 5000 production servers over a 6-month period have shown that although servers usually are not idle, the utilization rarely approaches 100%. Most of the time, servers operate at 10%~50% of their full capacity, leading to extra expenses on overprovisioning and thus extra total cost of acquisition [1]. Another problem is the narrow dynamic power range of servers. Even completely idle servers still consume about 70% of their peak power [2]. Therefore, keeping servers underutilized is highly inefficient from the energy consumption perspective.

Many techniques can improve energy efficiency, such as improvement of applications' algorithms, energy efficient hardware, Dynamic Voltage and Frequency Scaling (DVFS), terminal servers, and thin clients. Cloud computing mainly leverages the capabilities of the virtualization technology to address the energy inefficiency problem [3]. The virtualization technology allows cloud providers to create multiple virtual machine (VM) instances on a single physical server, thus improving the utilization of resources and increasing the return on investment. The reduction in energy consumption can be achieved by switching idle nodes to low-power modes (i.e., sleep or hibernation), thus eliminating the idle power consumption. Moreover, by using live migration [4], the VMs can be dynamically consolidated to the minimal number of physical nodes according to their current resources requirements.

However, virtualization also creates a new problem. One essential requirement of a cloud computing environment is providing reliable QoS defined in terms of service level agreements (SLA). Modern applications often experience highly variable workloads causing dynamic resources usage patterns. The consolidation of VMs can lead to performance degradation when an application encounters an increasing demand resulting in an unexpected rise of resources usage. This may lead to SLA violation—increasing response times, time outs, or failures. Overprovisioning may help to ensure SLA, but it leads to inefficiency when the load decreases. The optimal strategy is to timely adjust resources provisioning according to the actual demands of the application. The precondition of this approach is to find out the future workload.

The focus of this work is on improving the efficiency of resources provisioning by forecasting the load of various resources in cloud. We propose a multi-step-ahead load prediction method, KSwSVR, mainly based on statistical learning technology, support vector regression (SVR), which is suitable for the complex and dynamic characteristics of the cloud computing environment. To the best of our knowledge, it is the first time that SVR is used for load forecasting in cloud. KSwSVR integrates our improved SVR algorithm and Kalman smoothing technology. Experiments with public trace data have shown that, in comparison with AutoRegressive (AR), Back-Propagation Neural Network (BPNN), and standard SVR, KSwSVR always has the minimum prediction error. Furthermore, KSwSVR is very stable; that is, its prediction error increases quite slowly when the predicted steps increase. We also verified the broad adaptability of KSwSVR with real trace data of various resources related to network, CPU, memory, and storage systems. Based on the predicted results, a simple and efficient strategy is proposed for resource provisioning, considering the variations of prediction error and SLA levels. Finally, the usefulness of this method is demonstrated in a CPU allocation experiment. With the assistance of KSwSVR, dynamic provisioning strategy can save 17.20%~48.12% CPU capacities under different SLA levels, comparing with static provisioning.

The rest of this paper is organized as follows.  Section 2 describes the background and motivation.  Section 3 discusses the detailed design and implementation of the proposed approach and Section 4 presents the experimental evaluation.  Section 5 examines the related work and Section 6 makes the conclusion.

## 2. Background and Motivation

### 2.1. Definitions

#### 2.1.1. Load

The object processed by the entity. For different entities, load refers to different objects, such as user requests of web server, computing tasks of CPU, read/write requests of storage system, and *I/O* requests of external device.

#### 2.1.2. Utilization/Usage

The amount of system resources used by applications, usually expressed as the ratio of the used part to the total resource. Efficient utilization/usage means that applications use the resources allocated to them as fully as possible.

#### 2.1.3. QoS

Quality of service refer to a certain level of performance and availability of a service. It also covers other aspects which are outside the scope of this paper, such as security and reliability.

#### 2.1.4. SLA

Service level agreements, a series of goals obtained through negotiating between service providers and customers. Its purpose is to achieve and maintain a specific QoS. Typical parameters of cloud computing SLA include CPU and memory capacity, resource expansion speed and permissions, resource availability and reliability, application response time, communication delay, and security and privacy. It also defines the penalties that should be imposed when someone violates the relevant terms.

#### 2.1.5. VM Size

The quantity of each resource of virtual machine, such as CPU, memory, storage, bandwidth. It is usually a multidimensional variable, for example, “2core∗3GHz CPU, 2 G memory, 30 G disk, and 10 M bandwidth”.

### 2.2. Load in Cloud

With the proliferation of private and public cloud data centers, it is quite common today to lease virtual machines to host applications instead of physical machines. Cloud users typically pay for a statically configured VM size, irrespective of the actual resources consumption of the application (e.g., Amazon EC2). This charging mode is obviously unreasonable especially for applications with variable load. It is usually difficult for cloud users to figure out which size of VM is suitable for their applications as their loads are rarely constant. They certainly do not like to pay for the resources they hold but not use when load is light. Furthermore, they have to face the risk of performance degradation when the load is heavy. In addition, cloud providers, such as Amazon EC2, provide resources on a VM basis. VMs are added, released, or migrated according to variation of load. Each process involves significant overhead but does not bring any actual benefit. It would be highly desirable for cloud providers to provide dynamically finer-grained online scalable services that allocate resources according to application's demand that could encourage the customer to pay a higher price for better service compared to paying a flat fee based on the size of their VMs. Moreover, cloud providers can have the flexibility to dynamically optimize the resources allocation, improve resources utilization, and achieve maximum benefit.

Timely and dynamic fine-grained online scalability will greatly increase the pressure on management system to rapidly detect and resolve SLA violation problems. Typically, problem detection is done by specifying threshold tests. Examples include “Do ping response times exceed 0.5s?” and “Are HTTP operations greater than 12 per second on server?” Unfortunately, once the detection occurs, there is often little time to take corrective actions. In addition, if the load changes dramatically, there will be frequent SLA violations. It is desirable that the resources can be acquired earlier than the time when the load actually increases. We need a predictive solution instead of a reactive strategy. This outcome would be possible only if the future load can be predicted. According to the predictive value, we can prepare for retrieving upcoming idle resources, providing them to other users or converting them to energy saving mode in advance or we can add resources for the upcoming peak load in advance to ensure a stable QoS.

**Figure 1:**
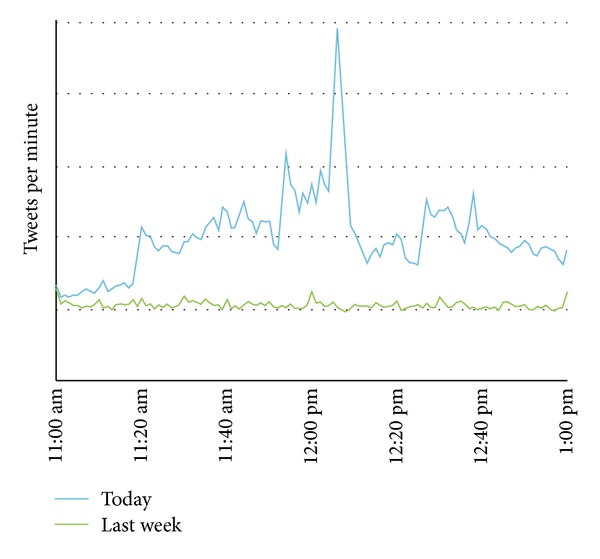
Load of Twitter on Obama's inauguration day.

However, load forecasting is difficult in cloud computing environment for the following reasons. First, most modern applications have fluctuant loads which lead to complex behaviors in resources usages as their intensity and composition change over time. For example, Figure 1 depicts a real-world scenario wherein Twitter experienced dramatic load fluctuations on Obama's inauguration day [5]. Such a load is very typical in modern commercial websites, and load forecasting for such application is not easy. Second, for security and privacy, cloud service providers are usually forbidden to access the internal details of the application. So, cloud management system cannot take advantage of the application's internal characteristics (e.g., a loop code indicates that resources usage will exhibit periodic similarity) to forecast load. For example, Niehorster et al. used sensors to read the behavior of application [6]. It is infeasible in most cases. Third, unlike traditional computing environment, in cloud, the external environment which the applications have to face is dynamic. Interference among applications hosted on the same physical machine leads to complex resources usage behaviors as they compete for various types of resources which are hard to strictly partition. For instance, in the exclusive nonvirtualized environment, an application with constant workload should have relatively stable resources demand. But in cloud where cohosted applications compete for the shared last level cache or disk *I/O* bandwidth, the usage of resources that can be strictly partitioned and allocated (e.g., CPU or memory) will likely fluctuate.

### 2.3. Support Vector Machine

Modern cloud computing datacenters is comprised of heterogeneous and distributed components, making them difficult to manage piecewise, let alone as a whole. Furthermore, the scale, complexity, and growth rate of these systems render any heuristic and rule-based system management approaches insufficient. It is also infeasible to forecast load by modeling the behaviors of the various applications and their relationships to each other. In response to these challenges, statistics-based techniques for building gray or black box models of applications load can better guide resources provisioning decision in cloud. Our study treats the load forecasting as a time series prediction problem and makes use of statistical learning method—support vector machine (SVM).

SVM was developed by Vapnik and used for many machine learning tasks such as pattern recognition, object classification, regression analysis, and time series prediction [7]. It is based on the structural risk minimization (SRM) principle which tries to control the model complexity as well as the upper bound of generalization risk. The principle is based on the fact that the generalization error is bounded by the sum of the empirical error and a confidence interval term that depends on the Vapnik-Chervonenkis (VC) dimension. On the contrary, traditional regression techniques, including traditional artificial neural networks (ANN) [8], are based on empirical risk minimization (ERM) principle, which tries to minimize the training error only. Furthermore, the learning process of ANN is quite complex and inefficient for modeling, and the choices of model structures and parameters are lack of rigorous theoretical guidance. So, it may suffer from overfitting or underfitting with ill-chosen parameters. In contrast, SVM has strict theory and mathematical foundation that do not have the problem of local optimization and dimensional disaster. It can achieve higher generalization performance especially for small samples set. It has a limited number of parameters to choose for modeling, and there exist fast and memory-efficient algorithms.

SVR is the methodology by which a function is estimated using observed data which in turn train the SVM. Its goal is to construct a hyperplane that lays close to as many of the data points as possible. This goal is achieved by arriving at the most flat function which ensures that the error does not exceed a threshold *ε*. Flatness is defined in terms of minimum norm whereas the error threshold is introduced as a constraint. Slack variables are introduced to deal with the situations where the above definition followed in the strict sense leads to an infeasible solution.

**Figure 2:**
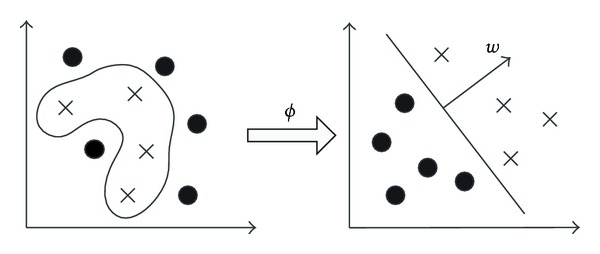
Mapping data from input space to feature space.

When performing time series prediction by SVM, each input vector *x*_*i*_ is defined as a finite set of consecutive measurements of the series. The output vector *y*_*i*_ contains the *x*_(*n*+1)_ observation, where *n* determines the amount of history data. Each combination (*x*_*i*_, *y*_*i*_) constitutes a training point. There are *N* such training points used for fitting the SVR. SVM is a linear learning machine. The linear function is formulated in the high dimensional feature space, with the form

(1) $$\begin{array}{c} f\left(x\right)=w\phi \left(x\right)+b, \end{array}$$

where *x* is nonlinearly mapped from the “input” space to a higher dimension “feature” space via mapping function *ϕ*; see Figure 2. To simplify the mapping, kernel function *K*(*x*_*i*_, *x*_*j*_) = 〈*ϕ*(*x*_*i*_), *ϕ*(*x*_*j*_)〉 is used. The most widely used kernel functions are

 linear: *K*(*x*_*i*_, *x*_*j*_) = *x*_*i*_^*T*^ *x*_*j*_
 polynomial: *K*(*x*_*i*_, *x*_*j*_) = (*γx*_*i*_^*T*^ *x*_*j*_+*r*)^*d*^, *γ* ≥ 0
 radial basis function (RBF): *K*(*x*_*i*_, *x*_*j*_) = exp⁡(−*γ*||*x*_*i*_−*x*_*j*_||^2^), *γ* ≥ 0
 sigmoid: *K*(*x*_*i*_, *x*_*j*_) = tanh(*γx*_*i*_^*T*^ *x*_*j*_ + *r*).

We choose the RBF kernel as it is easier to compute and has fewer parameters to adjust.

The goal is to find “optimal” weights *w* and threshold *b* as well as to define the criteria for finding an “optimal” set of weights. First is the “flatness” of the weights, which can be measured by the Euclidean norm (i.e., minimize||*w*||^2^). Second is the error *R*_emp_ generated by the estimation process of the value, also known as the empirical risk that is to be minimized. The overall goal is to minimize the regularized risk *R*_reg_ (using the *ε*-insensitive loss function) as

(2) minimize Rreg=12||w||2+Remp=12||w||2+C∑i=1nLε(yi,f(xi)),

where

(3) $$\begin{array}{c} L^{\varepsilon }\left(y_{i},f\left(x_{i}\right)\right)=\{\begin{array}{cc} \left|y_{i}-f\left(x_{i}\right)\right|-\varepsilon & \left|y_{i}-f\left(x_{i}\right)\right|\geq \varepsilon ,\\ 0 & \text{otherwise}, \end{array} \end{array}$$

*R*_emp_ in (2) is measured by the *ε*-insensitive loss function *L* ^*ε*^. *C* is the regularization constant determining the tradeoff between the empirical risk and regularized risk. It should be noted that both *ε* and *C* are user defined constants and are typically computed empirically. Introduction of the positive slack variables, *ζ* and ζ*, which, respectively, denote the errors above and below *ε*, leads (2) to the following constrained function:

(4) minimize  Rreg=12||w||2+C∑i=1n(ζi+ζi∗)subject  to f(xi)−wϕ(xi)−bi≤ε+ζi      wϕ(xi)+bi−f(xi)≤ε+ζi∗,                  ζi(∗)≥0.

From the implementation point of view, training SVM is equivalent to solving a linearly constrained quadratic programming (QP) problem with the number of variables equal to the number of training data points. The sequential minimal optimization (SMO) algorithm [9] is very effective in training SVMs for solving the regression estimation problem.

## 3. Approach

The main body of our multi-step-ahead load forecasting method is based on our improved *SVR*. Rather than giving the same consideration to the training data within a sliding window in standard SVR, our multi-step-ahead load forecasting strategy gives more *weight* to more “important” data.

In order to enhance prediction accuracy, the *Kalman Smoother* is used for data preprocessing. We argue that Kalman smoother is suitable for the cloud application's load estimation because it was originally developed to estimate time-varying states in dynamic systems. This approach essentially uses a filtering technique to eliminate the noise of resources usage signal coming from error of measurement technique while still discovering its real main fluctuations.

We give this method a name, *KSwSVR*.

### 3.1. Kalman Smoother (KS)

The Kalman filter [10] has been widely used in the area of autonomous or assisted navigation. One of the main advantages of the filter is that it can estimate hidden parameters indirectly from measured data and can integrate data from as many measurements as are available, in an approximately optimal way. The Kalman filter estimates the state *x* of a discrete-time controlled process which is governed by the linear stochastic difference equation:

(5) $$\begin{array}{c} x_{k}=Ax_{k-1}+Bu_{k-1}+w_{k-1}. \end{array}$$

With a measurement *z* that is

(6) $$\begin{array}{c} z_{k}=Hx_{k}+v_{k}, \end{array}$$

where *A* is a transform matrix from time step *k* − 1 to *k*. *u*_*k*−1_ represents a known vector. *B* is a control matrix. *H* is a matrix that presents the relation of *z*_*k*_ and *x*_*k*_. The random variables *w*_*k*−1_ ~ *N*(0, *Q*_*k*−1_) and *v*_*k*_ ~ *N*(0, *R*_*k*_) represent the process and measurement noise, respectively. They are assumed to be white and independent of each other. The Kalman filter estimates a process by using a form of feedback control: the filter estimates the process state and then obtains feedback in the form of (noisy) measurements. As such, the equations for the Kalman filter fall into two groups: *time update* (7) (predictor) and *measurement update equations* (8) (corrector). *K* is known as *Kalman Gain*. The time update projects the current state estimate ahead of time. The measurement update adjusts the projected estimate by an actual measurement at that time

(7) Time  update  x^k−=Ax^k−1+Buk−1, Pk−=APk−1AT+Q,

(8) Measurement  update   Kk=Pk−HT(HPk−HT+R)−1, x^k=x^k−+Kk(zk−Hx^k−), Pk=(I−KkH)Pk−.

The Kalman filter only considers *P*(*x*_*t*_ | *y*_0:*t*_) as the filtered estimate of *x*_*t*_ only takes into account the “past” information relative to *x*_*t*_. By incorporating the “future” observations relative to *x*_*t*_, we can obtain a more refined state estimate. That is why we choose the Kalman smoother as our noise reduction method. The Kalman smoother, which can be calculated from the Kalman filter results by recursions, estimates *P*(*x*_*t*_ | *y*_0:*T*_, *t* < *T*), taking into account both past and future information. It is also computationally attractive, due to its recursive computation, since the production of the next estimate only requires the updated measurements and the previous estimations.

In our scenario of cloud application load, there is no control input, so *u* = 0. The noisy measurement is of the state directly, so *H* = 1. We assume the state does not change from step to step, so *A* = 1. Given the existence of relatively accurate measurement tools, we set *Q* = 0.1 and *R* = 1. Therefore, we use the (5) and (6) as follows:

(9) $$\begin{array}{c} x_{k}=x_{k-1}+w_{k-1},\\ z_{k}=x_{k}+v_{k},\\ w\sim N\left(0,0.1\right),\;v\sim N\left(0,1\right). \end{array}$$

### 3.2. SVR with Weighted Training Data (wSVR)

Treating all data of a time series equally is clearly unreasonable. We should take advantage of the data according to their “importance”—usefulness of data for improving prediction accuracy.

In the time series of nonstationary system, the dependency between input variables and output variables gradually changes over time. Specifically, recent past data could provide more important information than the distant past data. This conclusion is also true in this paper's cloud scenario. One of the most powerful arguments is *locality of reference*, also known as *principle of locality* [11]. As one of the cornerstones of computer science, locality of reference was born from efforts to make virtual memory systems work well, which is a phenomenon of the same value or related storage locations being frequently accessed. But today, this principal has found application well beyond virtual memory and directly influenced the design of processor caches, disk controller caches, storage hierarchies, network interfaces, database systems, graphics display systems, human-computer interfaces, individual application programs, search engines, web browsers, edge caches for web-based environments, and computer forensics. Therefore, we have good reason to believe that cloud load would follow the law as well. Hence we take the same principle that gives more weight to the more important recent historical data of the load. The newer the data are, the more important they are.

**Figure 3:**
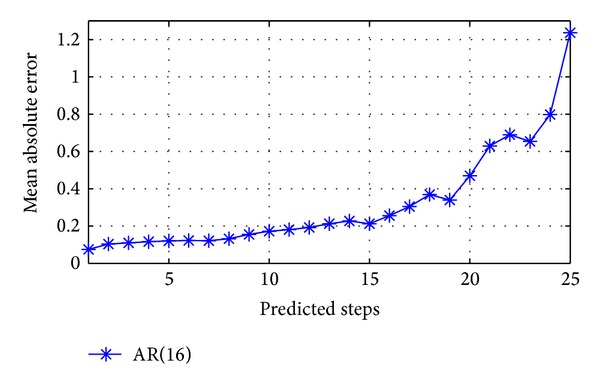
Relationship between MAE and predicted steps.

Another factor that influences the importance of data is their “credibility.” In our multi-step-ahead load forecasting, there are two types of data—measured data and predicted data. Measured data refer to the true historical resources usage information collected by the system monitor. Many popular monitor tools, such as the *top* for Linux and the *xentop* for Xen [3], are available for obtaining system information (e.g., usage of CPU, memory, network, and block device on host or VM). Monitor periodically collects system information for decision making. As mentioned before, cloud system is usually soft real time. Therefore, there is generally not a severe time constraint on monitoring period. Furthermore, too small monitoring granularity will bring more decision making costs and is not conducive to the improvement of system resource utilization. For example, once every 5 seconds is enough. Measured data are believed to have a high credibility (more important). Predicted data, the result of load forecasting algorithm, have lower credibility (less important) because any prediction algorithm has prediction error. A multi-step-ahead prediction can be achieved by running one-step-ahead prediction iteratively. Time series data related to once m-step-ahead prediction are $\left\{\ldots ,x_{t-2},x_{t-1},{\hat{x}}_{t},{\hat{x}}_{t+1},{\hat{x}}_{t+2},\ldots ,{\hat{x}}_{t+m-1}\right\}$ . The prediction of ${\hat{x}}_{t+m-1}$ is based on the series $\left\{\ldots ,x_{t-2},x_{t-1},{\hat{x}}_{t},{\hat{x}}_{t+1},{\hat{x}}_{t+2},\ldots ,{\hat{x}}_{t+m-2}\right\}$ , where only {…, *x*_*t*−2_, *x*_*t*−1_} are the measured data and $\left\{{\hat{x}}_{t},{\hat{x}}_{t+1},{\hat{x}}_{t+2},\ldots ,{\hat{x}}_{t+m-2}\right\}$ are predicted data. This process is based on a significant hypothesis that predicted data are assumed to be measured data when performance next step prediction. However, due to prediction error and dynamic feature of cloud, this hypothesis cannot be satisfied all the time when multi-step-ahead prediction carried out. Particularly, we need to address the accumulation of prediction errors. Every one-step-ahead prediction may cause an error. Therefore, using the former prediction results as the input data for next prediction will cause accumulation of errors.  Figure 3 depicts the relationship between prediction mean absolute error (MAE) and predicted steps, where the load series we used is collected from a real world *I/O* trace of an online transaction processing (OLTP) applications [12] and predictor is AR(16) [13]. It indicates that with the increase of predicted steps, the MAE is increasing drastically; that is, the predicted data's credibility is decreasing.

To sum up the above arguments, in multi-step-ahead load forecasting of cloud, the importance of input data series gradually increases and then decreases. The inflection point is between last measured data and first predicted data.

From (2), it can be observed that the performance of SVR is sensitive to the regularization constant *C*. A small value for *C* will underfit the training data because the weight placed on the training data is too small thus resulting in large value of prediction error. On the contrary, when *C* is too large, SVR will overfit the training data, leading to deterioration of generalization performance.

By using a fixed value of *C* in the regularized risk function, standard SVR assigns equal weights to all the *ε*-insensitive errors between the measured and predicted data, treating two types of data equally. For illustration, the empirical risk function in standard SVR is expressed by

(10) $$\begin{array}{c} R_{\text{emp}\_\text{std}}=C\sum_{i=1}^{n}\left(\zeta_{i}+\zeta_{i}^{*}\right). \end{array}$$

As discussed above, this is unreasonable. Thus, it is beneficial to place different weight on the *ε*-insensitive errors according to the importance of training data. So, we add a weight coefficient *w*_*i*_ to the regularization constant, translating the empirical risk function to

(11) $$\begin{array}{c} R_{\text{emp}\_w}=C\sum_{i=1}^{n}w_{i}\left(\zeta_{i}+\zeta_{i}^{*}\right),\\ w_{i}=\{\begin{array}{cc} f_{\text{measured}}\left(i\right) & i<t,\\ f_{\text{predicted}}\left(i\right) & i\geq t, \end{array} \end{array}$$

where *f*_measured_(*i*) is monotonically increasing function for measured data, while *f*_predicted_(*i*) is monotonically decreasing function for predicted data. *f*_∗_(*i*) may be a linear function, an exponential function, or others meeting the monotonicity requirement. Obviously, the choice of *f*_∗_(*i*) will affect the subsequent prediction accuracy. The optimal solution is taking into account characteristics of the data. This is another point worth studying.

### 3.3. Resources Provisioning Based on KSwSVR

Directly taking the predicted value as the final resources provisioning value may lead to unacceptable SLA violation since any prediction algorithm has an error range. Furthermore, as we can see in the latter experiment, even the same algorithm will have different prediction errors as the prediction object changes. That is, for different system resources (CPU, memory, or *I/O*) or the same resource at different time, allocating resources directly according to forecasting results cannot guarantee a stable QoS.

Therefore, we propose a simple resource allocation mechanism based on load forecasting for two considerations. First, load spike, which will lead to underprovisioning, is difficult to predict for any prediction algorithm especially in dynamic cloud. Second, in cloud computing model, the user's requirements of QoS also can change at any time. We need a flexible mechanism to deal with different SLA levels in this multitenant environment. The actual allocation value is computed as

(12) $$\begin{array}{c} x_{t}^{\text{alloc}}=s{\hat{x}}_{t}+\frac{1}{k}\sum_{i=t-1}^{t-k}d_{i},\\ d_{i}=\max\left\{0,x_{i}^{\text{use}}-x_{i}^{\text{alloc}}\right\}, \end{array}$$

**Figure 4:**
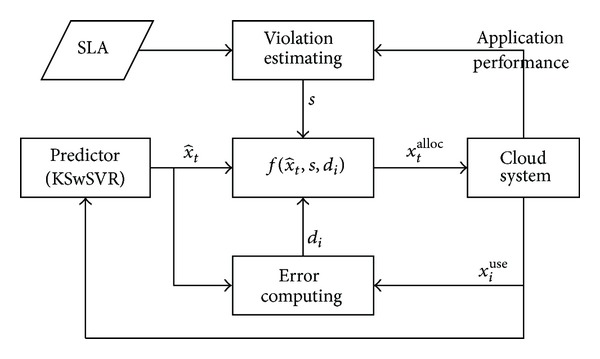
Decision-making process of resource provisioning.

*x*_*t*_^use^, ${\hat{x}}_{t}$ , and *x*_*t*_^alloc^ separately represent real resources usage, predicted value, and actual resources allocation value at time *t*. With *d*_*i*_, we use the information of underprovisioning in the last *k* periods, while ignoring overprovisioning. This allows system to quickly respond to load spike. *s* is an incremental coefficient which is highly correlated with QoS of cloud. Its value depends on the gap between the actual application performance and SLA. The greater the gap, the bigger the *s*. Bigger *s* means allocating more resources and fewer SLA violations. It is a proactive (KSwSVR) and QoS-driven (*s*) decision making process with a feedback (*d*_*i*_); shown in as Figure 4. Obviously, it is also applicable to other predictors.

## 4. Evaluation

In this section, the performance of KSwSVR will be evaluated by using various types of real-world trace data and comparing with other typical load forecasting technology. We prefer using public trace data rather than historical data generated by ourselves for the purpose of giving comparable and reproducible results.

### 4.1. Prediction Algorithm

In order to highlight the prediction performance of KSwSVR, two widely used prediction methods are chosen for comparison. They are typical representatives of linear prediction algorithm and machine learning technology—AR and BPNN. Meanwhile, standard SVR is also our comparison object.

#### 4.1.1. Linear Prediction—AR

Dinda and O'Hallaron [13] studied different linear load forecasting models including AR, moving average, autoregressive moving average, autoregressive integrated moving average, and autoregressive fractionally integrated moving average models. Their conclusion is that the simple AR model is the best model and is appropriate and sufficient to be used for load prediction.

AR is a basic linear time series prediction algorithm in which the current value can be represented by the sum of a linear combination of several previous values and an error *ε*. The general expression of AR(*p*) model can be denoted as (13), where {*x*_1,_ *x*_2_,…, *x*_*t*_} is the time series, *p* is the order of AR model, *φ* = (*φ*_1_, *φ*_2_,…, *φ*_*p*_) denotes the coefficients of AR model.

Consider

(13) $$\begin{array}{c} x_{t}=\sum_{i=1}^{p}\varphi_{i}x_{t-i}+\varepsilon_{t}. \end{array}$$

We adopt Dinda's recommendation that AR(16) is the best in consideration of both overhead and prediction accuracy.

#### 4.1.2. Machine Learning—BPNN

As SVR, ANN [8] is also a typical machine learning strategy in the category of regression computation. ANN is a powerful tool for self-learning, and it can generalize the characteristics of load by proper training. It is inherently a distributed architecture with high robustness and has been used in resources state prediction in the past. It is indicated by Eswaradass et al. [14] that the ANN prediction outperforms the Network Weather Service methods [15].

**Figure 5:**
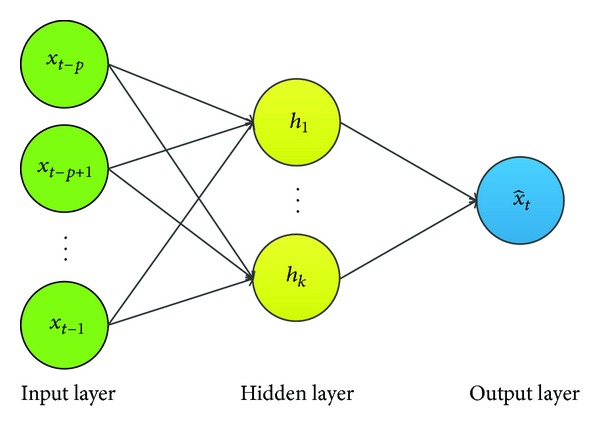
Standard multilayer feedforward neural network.

The structure of a standard multilayer feedforward neural network is in Figure 5. It consists of an input layer with input neurons [*x*_*t*−*p*_, *x*_*t*−*p*+1_,…, *x*_*t*−1_], a hidden layer with hidden neurons [*h*_1_, *h*_2_,…, *h*_*k*_], and an output layer with one output neuron ${\hat{x}}_{t}$ . Every node in a layer is connected to every other node in the neighboring layer. These connections are known as synapses. Each synapse is associated with a weight which is to be determined during training. During the training phase, the network is fed with input vectors, and random weights are assigned to the synapses. After presentation of each input vector, the network generates a predicted output ${\hat{x}}_{t}$ . The generated output is then compared with the actual output *x*_*t*_. The difference between the two is known as the error term.

The BPNN algorithm is the most popular and the oldest supervised learning feedforward neural network algorithm proposed by Rumelhart and Mcclelland [16]. The BPNN learns by calculating the errors of the output layer to find the errors in the hidden layers. The algorithm is highly suitable for the solution to problems in which no relationship is found between the output and inputs. Due to its flexibility and learning capabilities, BPNN has been successfully used in wide range of applications. Therefore, we chose it as one of the comparison object and empirically configure it with six input neurons and one hidden layer with ten hidden neurons, as considering both prediction overhead and accuracy.

#### 4.1.3. SVR and KSwSVR

Parameter configuration of standard SVR and KSwSVR in this work is as follows.

SVM-type:  *ε*-regression

SVM-kernel:  radial basis function (RBF)

Cost (C):  1, penalty parameter of the error term

Gamma:  0.0625, parameter of the RBF

Epsilon (*ε*):  0.1, *ε*-insensitive loss function

*f*_∗_(*i*):  linear function, for KSwSVR in (11).

It is worth emphasizing that we do not have tuned parameters of BPNN, SVR, and KSwSVR for specific predictions object, but set the same parameters for all trace data according to domain knowledge. Therefore, the experimental result of this paper is representative.

### 4.2. Experiment Setup

The implementation of KSwSVR is based on libsvm [17].

**Figure 6:**
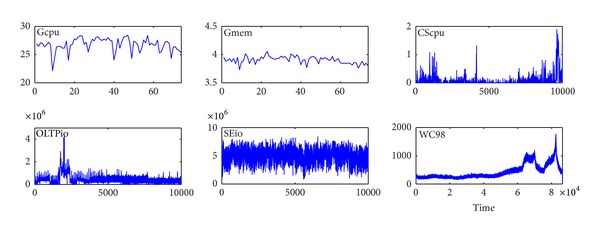
Trace data used in this work.

In order to highlight the adaptability of KSwSVR, we have collected public trace data of various type resources, involving the network, CPU, memory, and storage systems (see Figure 6).

(*1) Gcpu/Gmem*. 7 hours of CPU and memory usage data in Google cluster (TraceVersion1) [18]. For confidentiality reasons, the consumption of CPU and memory is obscured using a linear transformation before release. We randomly selected a long duration job with jobID 1485896354.

(*2) CScpu*. CPU load trace of a big memory compute server in the CMCL (Computers, Media, and Communication Laboratory) at Carnegie Mellon University [19]. The data is the number of processes that are running or are ready to run, which is the length of the ready queue maintained by the scheduler.

(*3) OLTPio*. Storage system data request rate derived from *I/O* trace of an OLTP applications running at large financial institutions [12].

(*4) SEio*. Storage system data request rate derived from *I/O* trace of a search engine [20].

(*5) WC98*. Client request rate observed in World Cup 98 web servers, from 1998-06-22:00.00 to 1998-06-22:23.59 [21].

First, we have evaluated the forecast performance of KSwSVR. The first 2000 points of each trace is used as our experiment data and translated to mean value of five intervals, except Google cluster data for its limited amount. As previously mentioned, our prediction work is for timely and dynamic fine-grained online scalability of cloud, so that the overhead of each prediction would be as small as possible. In order to ensure forecasting speed, six training data were used each time in BPNN, standard SVR, and KSwSVR in our experiment.

Then, by simulating dynamic CPU allocation, we have shown the high efficiency of KSwSVR in resources provisioning.

### 4.3. Experimental Results

#### 4.3.1. Prediction Accuracy Evaluation

Before the training process begins, data normalization is performed by linear transformation as

(14) $$\begin{array}{c} x_{i}^{n}=\frac{x_{i}-x_{\min}}{x_{\max}-x_{\min}}, \end{array}$$

where *x*_*i*_^*n*^ and *x*_*i*_ represent the normalized and original measured data, respectively, and *x*_min⁡_ and *x*_max⁡_ represent the minimum and maximum value among the original measured data, respectively.

The evaluation of prediction performance is based on the mean absolute error (MAE) which is a widely used error metric for evaluating results of time-series forecasting, as shown in (15).

(15) $$\begin{array}{c} \text{MAE}=\frac{1}{n}\sum_{i=1}^{n}\left|{\hat{x}}_{i}-x_{i}\right|, \end{array}$$

where ${\hat{x}}_{i}$ and *x*_*i*_ represent the predicted and original data, respectively, and *n* is the number of predicted data points.

**Figure 7:**
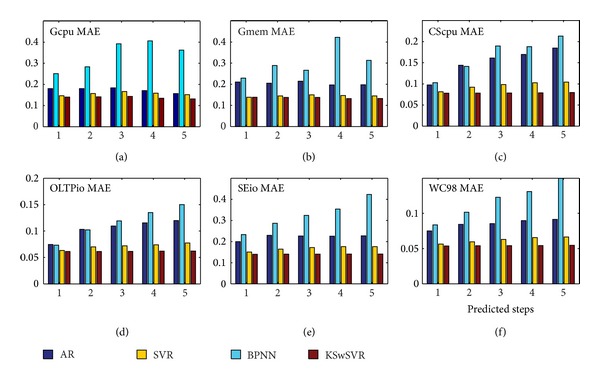
MAE comparison.

The detailed experimental results are shown in Figure 7. Because the total number of samples is limited, performance trend feature of Google's two traces fluctuate slightly. But we can still reach this conclusion: KSwSVR has the best prediction accuracy, followed successively by standard SVR and AR and BPNN.

AR, as a typical representative of the linear prediction technology that cannot be well adapted to the nonlinear regression problem, has a relatively high prediction error. Furthermore, when data have high volatility (e.g., CScpu and OLTPio), the error cumulative effect of AR is quite obvious. This makes it unsuitable for multi-step-ahead long-term load forecasting in cloud.

As an excellent machine learning technology, the neural network should have a better nonlinear regression performance. However, as mentioned before, timely and dynamic fine-grained online scalability of cloud needs a fast and efficient load forecasting algorithm. For this reason, the training data set for a predicted data should not be too large. So, suffering from this restriction, the performance of BPNN deteriorates significantly, and its prediction accuracy is even lower than that of AR. Its error cumulative effect is also large.

In contrast, the theory foundation of SVM determines its excellent performance in the face of small training sample set. For all six traces, SVR and KSwSVR have always maintained a relatively high prediction accuracy and stability and their error cumulative effect is quite slight.

**Figure 8:**
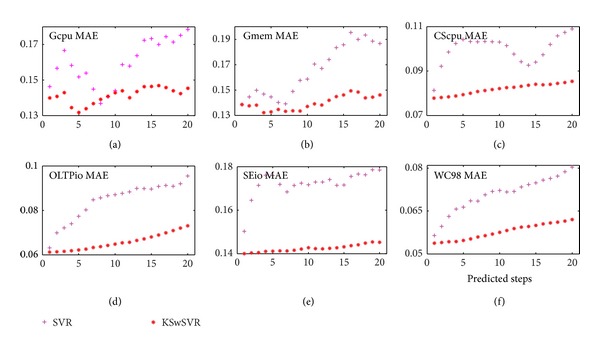
KSwSVR versus standard SVR.

We further compared KSwSVR and standard SVR with more predicted steps; see Figure 8. Benefiting from Kalman smoother and weight technology, KSwSVR outperforms standard SVR all the time, except two points of Gcpu for its small samples set. Furthermore, the prediction error of KSwSVR increases quite more slowly as predicted steps increases. That means its prediction accuracy is very stable. In contrast, the performance of standard SVR fluctuates with predicted steps. Specifically, improved MAE of KSwSVR is in Table 1.

#### 4.3.2. Computational Costs

As mentioned earlier, SVM is a relatively new machine learning method that optimizes model on training data. Because dot products in feature space can be represented by kernel functions, the transformation from input space to feature space is implicit. Training SVM is converted to solving a linearly constrained QP problem. That greatly reduces the computational complexity.

Moreover, the use of SMO to solve the SVM QP problem can further accelerate the training speed. SMO avoids the numerical QP optimization steps and requires no extra matrix storage at all [9].

Based on the above theories, we believe that the KSwSVR based on SVM should be efficient. With four traces in Figure 6, we have compared the temporal costs of AR, BPNN, standard SVR, and KSwSVR on the same computing platform. The choices of experimental parameters are the same as before, except that 10,000 data points of each trace are used as test data.

Experimental results, in Table 2, show that the two algorithms based on SVR have obvious efficiency advantages. The computational cost of KSwSVR increases slightly compared with that of standard SVR. However, as analyzed above, the introduction of the Kalman smoother significantly improves the forecasting performance. It is worthwhile. For any trace data, the total computational cost of KSwSVR is only about 0.7 seconds, which accounts for approximately 2% of the cost of AR (about 29 s) or 0.17% of the cost of BPNN (about 410 s). The algorithm complexity of BPNN determines its high computational cost. As a linear time series prediction algorithm, AR should have low temporal cost. However, in order to achieve acceptable prediction accuracy, AR requires more training samples each time that increases the computational cost. Since all data are processed as time series, the temporal costs of different types of traces are almost equal for the same prediction algorithm. That is, the computational cost of load forecasting algorithm is independent of the type of load. Thus, we can conclude that the KSwSVR has a relatively lower computational cost and is very suitable for online load forecasting. It can effectively support the timely and dynamic fine-grained scalability for real-time applications.

#### 4.3.3. Dynamic CPU Allocation Based on KSwSVR

We demonstrated the advantage of accurate load forecasting for resources provisioning with a real CPU load trace, CScpu [19]. To be clearer, we only use its first half in this experiment. Static CPU allocation with fixed allocation value was compared with our dynamic CPU allocation based on KSwSVR.

**Figure 9:**
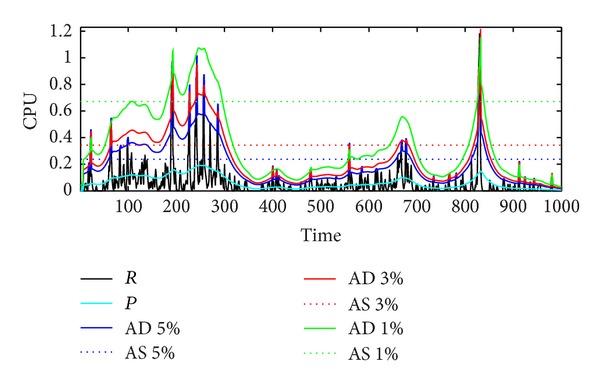
CPU allocation of dynamic/static strategy. *R*—real usage; *P*—predicted value; AD/AS—actual dynamic/static allocation value; *x*%: SLA violation rate.

We assume that SLA only requires no CPU underprovisioning. Any SLA violation rate can be achieved by adjusting incremental coefficient *s* in (12). The detailed experimental results are shown in Figure 9. Under the same SLA violation rate, the total CPU consumption of dynamic strategy is always less than static method. For example, when the SLA violation rate is 5%, dynamic CPU allocation strategy based on the KSwSVR (blue solid line) can reduce the CPU consumption by 17.20%, compared with the static CPU allocation strategy (blue dashed line). Detailed data is shown in Table 3. With the decrease of SLA violation rate, the benefit produced by KSwSVR becomes more significant. Specifically, the total allocated CPU amount even reduces by nearly half when SLA violation rate is 1%.

By cooperating with other technologies such as virtualization and DVFS, our method can effectively improve resources utilization and energy saving.

## 5. Related Work

We classify the related work into two categories: (1) forecasting technology in grid, cloud, and virtualization environment and (2) SVM/SVR related to our work. Other forecasting technology in computer science is not considered here.

The Network Weather Service (NWS) [15] is the most famous system designed to provide dynamic resources performance forecasts and has been mainly deployed as a grid middleware service. The predictive methods currently used include running average, sliding window average, last measurement, adaptive window average, median filter, adaptive window median, *α*-trimmed mean, stochastic gradient, and AR. Xu et al. [22] use fuzzy logic to model and predict the load of virtualized web applications. The VCONF project has studied using reinforcement learning combined with ANN to automatically tune the CPU and memory configurations of a VM in order to achieve good performance for its hosted application [23]. This solution is specifically targeted for only the CPU resources. Roy et al. [24] used a second order autoregressive moving average method filter for workload forecasting in cloud. Jiang et al. [25] proposed a self-adaptive prediction solution to enable the instant cloud resources provisioning. They employ a set of different prediction techniques as the base predictors, including moving average, AR, ANN, SVM, and gene expression programming. For the sake of well handling of the prediction task, a prediction ensemble method was proposed to combine the power of individual prediction techniques. One of the characteristics of this method is the large amount of calculation. It is not suitable for fine-grained online forecasting. In [26], PRESS first employs signal processing techniques (Fast Fourier Transform) to identify repeating patterns called signatures that are used for its predictions. If no signature is discovered, PRESS employed a statistical state-driven approach (discrete-time Markov chain) to capture short-term patterns of load. Experimental results show that its accuracy is similar to AR. Based on similar characteristics of web traffic, Caron et al. [27] proposed a pattern matching algorithm to identify closest resembling patterns similar to the present resources utilization pattern in a set of past usage traces of the cloud client. The resulting closest patterns were then interpolated by using a weighted interpolation to forecast approximate future values that were going to follow the present pattern. However, their approach has two problems. Firstly, searching for similar patterns each time over the entire set of historical data is inefficient. And secondly, it may lead to overspecialization, thus turning out to be ineffective. Islam et al. [28] explored Error Correction Neural Network (ECNN) and linear regression to make prediction on future resources requirement in the cloud. The authors also mentioned that it is also possible to accommodate other learning methods (e.g., SVM) for prediction of future resources usage. Bey et al. [29] presented a modeling approach to estimating the future value of CPU load. This modeling prediction approach uses the combination of Adaptive Network-based Fuzzy Inference Systems (ANFIS) and the clustering process applied on the CPU load time series. Liang et al. [30] presented a long-term prediction model applying Fourier transform to exploit the periods of the CPU waves and using tendency-based methods to predict the variation. Wu et al. [31] adopted an Adaptive Hybrid Model (AHModel) for long-term load prediction in computational grid. It is an improvement of their previous work HModel [32]. Both are based on AR and confidence interval estimations. However, the prediction range of AHModel is limited to 50-step-ahead. Moreover, AHModel cannot predict the load variation very well, especially the variation around the peak points.

SVM is widely used in financial data prediction, general business applications, environmental parameter estimation, electric utility load forecasting, machine reliability forecasting, control system, and signal processing [33]. As a relatively new forecasting technology, its application related to our work is comparatively rare at present. Prem and Raghavan [34] directly used SVM to forecast resources derived from NWS. SVR was used to build models predicting response time given a specified load for individual workloads cohosted on shared storage system in [35]. SVR has also been used to model power consumption as a function of hardware and software performance counters in [36]. Kundu et al. [37] proposed to use SVR and ANN to model the relationship between the resources provisioning to a virtualized application and its performance. Such a model can then be used to predict the resources need of an application to meet its performance target. But, their work was based on one assumption that application has static workloads and its behavior is stable. Obviously this assumption will severely restrict the feasibility of their method in dynamic cloud environment. Moreover, their offline performance modeling cannot timely respond to changes of environment and load. Niehorster et al. [6] used SVM to enable the SaaS (software as a service) provider to predict the resources requirements of an application. Different from our method, theirs is coarse-grained and offline. In addition, feature extraction of their work needs to know the application's internal detail, but this is generally prohibited in cloud.

## 6. Conclusion and Future Work

In order to achieve efficient resources provisioning in cloud, we propose a multi-step-ahead load prediction method, KSwSVR, based on statistical learning technology which is suitable for the complex and dynamic characteristics of the cloud computing environment. It integrates an improved support vector regression algorithm and Kalman smoothing technology and does not require access to the internal details of application. The improved SVR gives more weight to more “important” data than standard SVR, using the historical information more reasonably. Kalman Smoother is employed to eliminate the noise of resources usage data coming from measurement error. Public trace data of various resources were used to verify the excellent prediction accuracy, stability, and adaptability. In comparison with AR, BPNN, and standard SVR, KSwSVR always has the minimum prediction error facing every type of resources and different predicted steps. This statistical learning-based approach is not designed for a specific forecast object, so we believe it will exhibit outstanding performance when faced with various subjects (job, application, VM, host, and cloud system) and resources (computing, storage, and network). Subsequently, based on the predicted results, a simple and efficient strategy is proposed for resource provisioning, considering the variations of prediction error and SLA levels. CPU allocation experiment has shown that accurate load forecasting could significantly reduce resources consumption while ensuring QoS. It is beneficial to improve resources utilization and energy saving. We plan to integrate this method into an automated cloud resources management system in future work.

## Figures and Tables

**Table 1:** Improvement of prediction error.

Trace Data	Gcpu	Gmem	CScpu	OLTPio	SEio	WC98
MAE Improvement	12.9%	17.9%	22.0%	28.1%	20.8%	22.5%

**Table 2:** Comparison of total computational costs (*s*).

Trace Data	AR	BPNN	SVR	KSwSVR
CScpu	29.1467	409.5801	0.4287	0.6549
OLTPio	29.0108	410.7172	0.4480	0.7548
SEio	29.4803	411.9297	0.4308	0.6627
WC98	29.3089	409.8534	0.4415	0.7111

**Table 3:** Comparison of total CPU consumption.

Incremental coefficient (*s*)	3.0	3.8	5.6
SLA violation rate	5%	3%	1%
(Static-dynamic)/static	17.20%	29.81%	48.12%
